# The genus *Catathelasma* (Catathelasmataceae, Basidiomycota) in China

**DOI:** 10.3897/mycokeys.62.36633

**Published:** 2020-02-03

**Authors:** Zai-Wei Ge, Jian-Yun Wu, Yan-Jia Hao, Qingying Zhang, Yi-Feng An, Martin Ryberg

**Affiliations:** 1 CAS Key Laboratory for Plant Diversity and Biogeography of East Asia, Kunming Institute of Botany, Chinese Academy of Sciences, Kunming 650201, China Kunming Institute of Botany, Chinese Academy of Sciences Kunming China; 2 University of Chinese Academy of Sciences, Beijing, China University of Chinese Academy of Sciences Beijing China; 3 School of Horticulture, Anhui Agricultural University, Hefei 230036, China Anhui Agricultural University Hefei China; 4 Industrial Crops Research Institute, Yunnan Academy of Agricultural Sciences, Kunming, China Industrial Crops Research Institute, Yunnan Academy of Agricultural Sciences Kunming China; 5 Systematic Biology, Department of Organismal Biology, Uppsala University, Norbyvägen 18D, 75236 Uppsala, Sweden Uppsala University Uppsala Sweden

**Keywords:** Catathelasmataceae, Biannulariaceae, Tricholomataceae, taxonomy, ectomycorrhizal fungi, new taxa

## Abstract

Two new species, *Catathelasmalaorentou* and *C.subalpinum*, are described on the basis of morphological characters, phylogenetic evidence, host preferences and geographic distributions. A taxonomic key to the known species in China is also provided to facilitate identification. Based on samples from temperate Asia, Europe and North America, the phylogeny of *Catathelasma* was reconstructed using the internal transcribed spacer (ITS) region, the large subunit (LSU) of the ribosomal DNA and the translation elongation factor 1-α (TEF1).The phylogenetic results showed that *Catathelasma* contains two monophyletic clades: the /subalpinum clade and the /imperiale clade. The Asian species *C.laorentou* and *C.subalpinum* are closely related to the North American *C.* sp. (labelled as *C.ventricosum* in GenBank) in the /subalpinum clade, whereas *C.imperiale* and *C.singeri* are closely related in the /imperiale clade.

## Introduction

*Catathelasma* Lovejoy is the type genus of the mushroom family *Catathelasmataceae* Wasser (Wasser 1985; Sánchez-García et al. 2016). This genus was erected by Lovejoy (1910) based on the type species *C.evanescens* Lovejoy. Morphologically, species within this genus have distinct tricholomatoid basidiomes, decurrent to adnate to sinuate-adnexed lamellae, double annulus, white spores that are oblong, smooth, amyloid and acyanophilic, bilateral to subregular lamella trama, firm and white context, hyphae with clamp connections and an ixocutis, ixolattice or cutis as pileipellis.

*Catathelasma* has long been regarded a member of the *Tricholomataceae* (Singer 1975, 1986), but Jülich (1982) established the family *Biannulariaceae* Jülich, based on *Biannularia* Beck, which had been synonymised with *Catathelasma* (Singer 1940). In 1985, Wasser established the *Catathelasmataceae* to contain the only member *Catathelasma* (Wasser 1985), and this family has recently been emended to also include *Callistosporium* Singer, *Guyanagarika*Sánchez-García et al. (2016), *Macrocybe* Pegler & Lodge, *Pleurocollybia* Singer and *Pseudolaccaria* Vizzini et al. (Sánchez-García et al. 2016).

*Catathelasma* contains four species: *C.evanescens*, *C.imperiale*, *C.singeri* and *C.ventricosum* (Kirk et al. 2008, Singer 1986). Species within this genus have been suggested to be ectomycorrhizal (Trappe 1962; Kohzu et al. 1999; Tedersoo and Smith 2013) and tend to be found in coniferous forests in northern temperate regions (Singer 1979). In China, collections of *Catathelasma* have long been regarded as belonging to *C.imperiale* or *C.ventricosum* (Ying and Zang 1994; Yuan and Sun 2013; Zang et al. 1996). During our studies of the ectomycorrhizal fungi associated with members of the Pinaceae, especially *Keteleeria* spp. in China (Ge et al. 2012), a few collections of *Catathelasma* with distinct ITS sequences from *Catathelasma* sequences in GenBank were encountered. Here, we used morphological observations and multilocus phylogenetic analyses to (i) clarify the species identity of *Catathelasma* specimens in China and (ii) examine the phylogenetic relationships of *Catathelasma* species. We also took into account the geographic isolation and the host associations of the Chinese collections.

## Materials and methods

### Collections and morphological studies

*Catathelasma* specimens were collected in western and south-western China (Yunnan, Sichuan, Gansu and Tibet) and deposited in the Herbarium of Cryptogams, Kunming Institute of Botany, Chinese Academy of Sciences (HKAS). Herbarium materials identified as *Catathelasmaevanescens* Lovejoy and *Catathelasmasingeri* Mitchel & A.H. Sm were loaned from Denver Botanic Garden, Sam Mitchel Herbarium of Fungi (DBG). Voucher information of the specimens and GenBank (Benson et al. 2017) accession numbers are detailed in Table 1.

**Table 1.** Taxa, vouchers, geographic origin and GenBank accession numbers of DNA sequences of *Catathelasma* and outgroups used in this study. New sequences generated in this study are given in bold. * indicates the type collection.

Taxon	Voucher	Geographic origin	GenBank accession number		
			ITS	LSU	TEF1
*Catathelasmasingeri*	DBG-F-006151	USA: Colorado	**MK909090**	**MK909109**	**MK909079**
*C.singeri*	DBG-F-021378	USA: Colorado	**MK909091**	**MK909110**	**MK909078**
	DBG-F-021747	USA: Colorado	**MK909092**	**MK909111**	**MK909080**
*C.singeri* as *ventricosum*	PBM 2403 (AFTOL-ID 1488)	USA: Washington	DQ486686	DQ089012	N/A
*C.imperiale*	HKAS 84299 (Z. W. Ge 3461)	China: Tibet	**MK909094**	**MK909112**	**MK909081**
	HKAS 84315 (Z. W. Ge 3477)	China: Sichuan	**MK909096**	**MK909113**	**MK909083**
	HKAS 79952 (X. B. Liu 251)	China: Sichuan	**MK909095**	**MK909114**	**MK909084**
	HKAS 76511 (X. T. Zhu 662)	China: Gansu	**MK909093**	**MK909115**	**MK909082**
	TAA176551	Canada: Newfoundland	N/A	AM946417	N/A
	UPS F-173429	Sweden: Uppland	**MK909097**	**MK909116**	**MK909085**
	UPS F-120619	Sweden: Hälsingland	**MK909098**	N/A	N/A
	TUB 011562		N/A	DQ071743 DQ071835	N/A
	LL_128		KX008987	N/A	N/A
	KM55154	UK: England	GQ981498	N/A	N/A
*C.* sp. as *imperiale*	DAOM225247		KP255468	AF261402	KP255475
	11CA01A	USA: California	N/A	N/A	KC816900
*C.* sp. as *ventricosum*	DAOM221514		KP255469	AF261401	N/A
	TRTC156545	Canada: Quebec	JN020996	N/A	N/A
	Mat3	Canada: Quebec	JN020995	N/A	N/A
	OSC 66879	USA: Pacific Northwest	EU669305	EU669331	N/A
	SMI349	Canada: British Columbia	HQ650727	N/A	N/A
	TAA176473	Canada: Newfoundland	N/A	AM946418	N/A
*C.laorentou*	HKAS 84458 (Z. W. Ge 3620)	China: Yunnan	**MK909106**	**MK909117**	**MK909086**
	HKAS 92245 (Z. W. Ge 3765)	China: Yunnan	**MK909103**	**MK909118**	**MK909087**
	*HKAS 105984 (Z. W. Ge 4070)	China: Yunnan	**MK909107**	N/A	N/A
	HKAS 71264 (T. Guo 368)	China: Yunnan	**MK909105**	**MK909119**	**MK909088**
	HKAS 78582 (L. H. Han 23)	China: Yunnan	**MK909108**	**MK909120**	N/A
	HKAS 76346 (Y. J. Hao 688)	China: Sichuan	**MK909102**	N/A	N/A
	HKAS 81166 (J. Qin 728)	China: Yunnan	**MK909104**	N/A	N/A
*C.subalpinum*	HKAS 70091 (Q. Cai 495)	China: Yunnan	**MK909100**	**MK909123**	**MK909089**
	*HKAS 67751 (J. Qin 65)	China: Yunnan	**MK909099**	**MK909121**	N/A
	HKAS 69920 (L. P. Tang 1459)	China: Yunnan	**MK909101**	**MK909122**	N/A
**Outgroups**					
*Callistosporiumgraminicolor*	PBM 2341 (AFTOL-ID 978)		DQ484065	AY745702	GU187761
*Callistosporiumluteoolivaceum*	DUKE-JM99124		N/A	AF261405	KP255477
	MSM#004		KJ101607	N/A	N/A

Morphological character descriptions were taken from field notes and colour images of the material, with colour names and codes following Kornerup and Wanscher (1978). Microscopic character observations followed published treatments on *Catathelasma* species (Mitchel and Smith 1978). Dried material was mounted in 5% aqueous (w/v) potassium hydroxide (KOH) under a Leica DM2500 microscope (Leica, Bensheim, Germany) and pileal structure, basidiospores and basidia were observed and measured in 5% KOH with 0.5% aqueous Congo Red (w/v). Melzer’s reagent was used to test the amyloidy of basidiospores. The length and width of at least 20 mature basidiospores from each specimen were measured in side view. Dimensions for basidiospores are reported as (a–) b–c (–d) and the abbreviation [n/m/p] indicates n basidiospores measured from m basidiomes of p collections. The range b–c contains a minimum of 90% of the measured values, with extreme values (a and d) presented in parentheses. Quotient of length and width (Q), average quotient (Qav) and standard deviation were calculated.

### DNA extraction, PCR and sequencing

Genomic DNA was extracted from dry specimens using the modified cetyltrimethylammonium bromide (CTAB) method (Doyle and Doyle 1987). Briefly, approximately 10 mg tissue was ground into a fine powder in liquid nitrogen in a 1.5 ml Eppendorf tube using a plastic pestle, and 500 μl of an extraction buffer (2 × CTAB) were added. The mixture was incubated at 60 °C for 1.5 h, with 0.2% ß-mercaptoethanol added prior to the extraction. Phenol-chloroform-isoamyl alcohol (25:24:1) were used to remove any proteins and polysaccharides and DNA was precipitated by adding 400 μl isopropanol to the aqueous phase. The DNA pellet was washed in 400 μl 70% ethanol and air-dried, then suspended in 80 μl TE (pH 8.0).

PCR amplification was performed following Ge et al. (2014) on an ABI 2720 Thermal Cycler (Applied Biosystems, Foster City, CA, USA). Primers used to amplify the internal transcribed spacer (ITS) region and the large subunit (LSU) of the ribosomal DNA and translation elongation factor 1-α (TEF1) were ITS1F/ITS4, LR0R/LR5 and 983F/1567R, respectively (Gardes and Bruns 1993; Matheny 2005; Rehner and Buckley 2005). Polymerase chain reaction (PCR) parameters follow those of Ge et al. (2014). PCR products were purified using a QIAquick PCR purification kit (Qiagen Science, USA) and sent to Kunming Shuoqing Biotech Ltd. (Kunming, China) for sequencing. Both directions were sequenced to improve accuracy. Sequencing primers were the same as the initial PCR primers. Sequence chromatograms were inspected and contigs assembled using Seqman version 5.01 (DNA STAR Package; DNAStar, Madison, WI, USA). The sequences produced in this study were deposited in GenBank with accession numbers MK909078–MK909123.

### Sequence alignment and phylogenetic analyses

DNA sequences of ITS, LSU and TEF1 were independently aligned with MAFFT v6.8 (Katoh et al. 2009) with manual adjustments and the concatenated datasets were manually constructed. Sequences of *Catathelasma* species, generated for this study and those of the genus that are available in GenBank, were included. *Callistosporiumgraminicolor* Lennox and *Callistosporiumluteo-olivaceum* (Berk. & M.A. Curtis) Singer were designated as outgroups based on previous phylogenetic studies (Ammirati et al. 2007; Sánchez-García et al. 2016). The datasets were then analysed using RAxML version 7.2.3 (Swofford 2002) and MrBayes v3.1.2 (Ronquist and Huelsenbeck 2003) for Maximum Likelihood (ML) and Bayesian Inference (BI), respectively. ML analyses were performed with 1000 bootstrap replicates, setting GTRGAMMAI as the selected model; and BI analyses were conducted with default parameters, except setting generations to 5 million and sampling every 1000^th^ generation. As selected by MrModeltest v2.3 (Nylander 2004), rates = gamma, nst = 2 was set for ITS dataset and rates = gamma, nst = 6 were set for LSU and TEF1, respectively. Since the average standard deviation of split frequencies converged (< 0.01) after 1 million generations, the first 25% of the sampled Bayesian trees (1251 trees) of the analysis were discarded as the burn-in. As no significant incongruence was observed using bootstrap values above 70% as threshold, we incorporated the ITS, LSU and TEF1 sequences into a concatenated dataset and performed the ML and BI analyses and partitioned the dataset by gene, as mentioned above. Final alignments were deposited in TreeBASE (http://www.treebase.org) under accession number S24480.

## Results

### Phylogeny and species recognition

**Figure 1.**
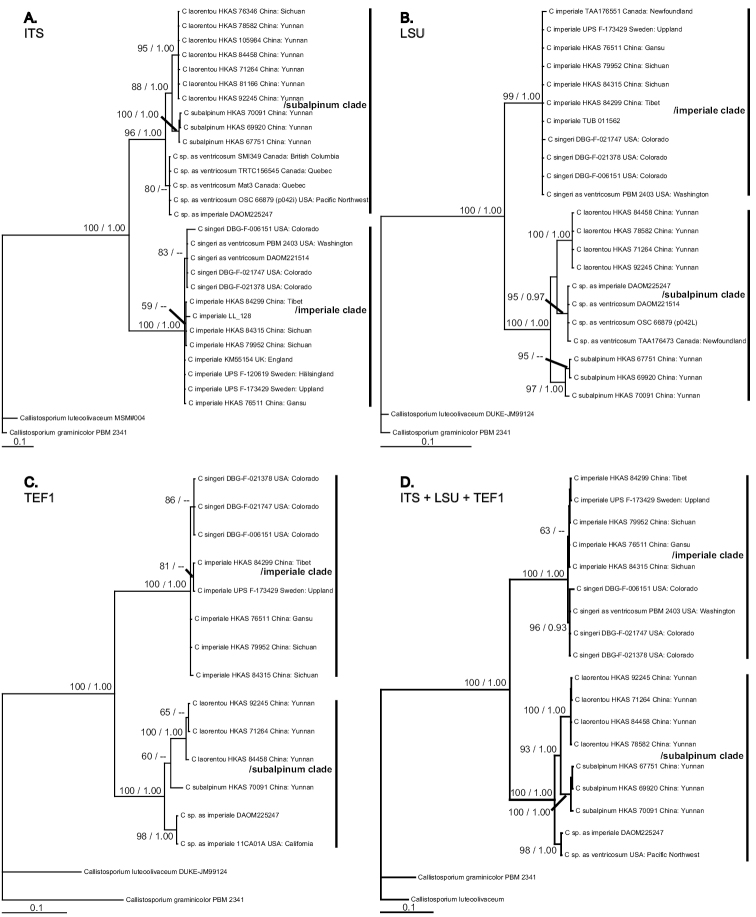
Bayesian phylogenies of **A**ITS**B** 28S **C**TEF1 and **D**ITS+28S+TEF1 concatenated sequences for *Catathelasma* species. Maximum likelihood bootstrap support and Bayesian posterior probabilities are indicated by values above branches.

Forty-six new ITS, LSU and TEF1 sequences were generated for *Catathelasma* species and deposited in GenBank (Table 1). The alignments of the ITS, LSU and TEF1 sequences were 708, 861 and 582 characters in length after trimming, respectively. ML and BI analyses produced consistent monophyletic clades and congruent phylogenies (Fig. 1).

Besides four Chinese collections that were confirmed to be conspecific with *C.imperiale*, sequences generated from other specimens collected in south-western China formed two monophyletic clades here described as *C.laorentou* and *C.subalpinum*, respectively (Fig. 1); each clade was well supported by both ML and BI in the ITS, LSU, TEF1 and concatenated trees (Fig. 1), except that in the TEF1 phylogeny, *C.subalpina* is only represented by a single sequence.

As revealed by the analyses of the different genetic markers and concatenated dataset (ITS, LSU, TEF1 and the combined dataset), the genus *Catathelasma* comprises two monophyletic clades: the /imperiale clade and /subalpinum clade (Fig. 1). Within the /subalpinum clade, *C.laorentou* appears to be sister to *C.subalpinum* and these Asian species jointly form the sister clade to the North American *C.* sp. (Fig. 1D, labelled as *C.ventricosum* in GenBank).

The /imperiale clade included the northern-temperate-region distributed *C.imperiale* and the North American species *C.singeri*. The ITS, TEF1 and concatenated sequences suggest that *C.singeri* represents a monophyletic clade within or close to *C.imperiale* (Fig. 1). In contrast to the /subapinum clade, the inter-species relationships within the /imperiale clade are not fully resolved: *C.singeri* is supported by the ML analyses, but not strongly supported by the BI tree (Fig. 1), although ITS sequences of *C.singeri* are only 94% (599/635)–-95% (542/570) similar to those of *C.imperiale*.

### Taxonomy

Considering the strong statistical support as monophyletic groups and the morphological differences, as well as their host preferences (see below), *C.laorentou* and *C.subalpinum* are described as new species.

#### Catathelasma imperiale

Taxon classificationFungiAgaricalesTricholomataceae

(P. Karst.) Singer

333E48E2-E1C9-5334-A469-ADF2F1A5CF89

Fig. 2C, D

##### Description.

Pileus 8–15 cm broad, hemispherical, convex to plano-convex, later expanded with decurved margin, sometimes depressed to funnel-shaped, smooth, dry to slightly viscid, greyish-brown, reddish-brownish or brown. Lamellae adnate to slightly decurrent, white to off-white when young, whitish to cream when mature, thick, 7–15 mm in height, with 1–2 series of lamellulae; edge smooth, grey to dark brown. Stipe 5–10 × 1.8–3.0 cm, fusiform, attenuate downwards, straight or curved, firm, with double annulus in which the lower annulus is often gelatinous and the upper annulus is membranous, with white to whitish upper surface and grey to brown lower surface. Context firm, white, not changing colour when cut; smell and taste farinaceous. Spore print white.

Basidiospores [60/3/3] 10–14.5 × 4.5–6 μm, hyaline in KOH, amyloid, congophilous, smooth, oblong to subcylindrical in frontal view, subcylindrical to somewhat inequateral in side view, thin-walled, without germ pore. Basidia 35–48 × 7–10 μm, 4-spored, narrowly clavate, hyaline; sterigmata up to 5 μm long. Cheilocystidia basidiole-like, with yellow to brown contents. Pleurocystidia absent. Lamella trama bilateral, composed of more or less parallel to interwoven hyphae. Oleiferous hyphae present in both lamella and pileus trama. Pileipellis a thick ixocutis of loosely interwoven cylindrical, 2–8 μm wide gelatinised hyphae, interspersed with oleiferous hyphae. Clamp connections present, common.

##### Ecology.

Ectomycorrhizal, solitary or scattered, in forests dominated by *Picea* spp. or *Abies* spp.

##### Specimens examined.

China. Gansu Province: Gannan city, Diebu, Wabagou, alt. 2700 m, 12 August 2012, X. T. Zhu 662 (HKAS 76511), under *Picea* sp.; Sichuan Province: Gangzi prefecture, Dege, Manigange, alt. 4200 m, 9 August 2013, Z. W. Ge 3477 (HKAS 84315), under *Piceaasperata* Mast.; same locality and date, X. B. Liu 251 (HKAS 79952); Tibet: on the way from Bangda to Changdu, 6 August 2013, Z. W. Ge 3461 (HKAS 84299), alt. 3980 m, under *Piceaasperata*.

**Figure 2.**
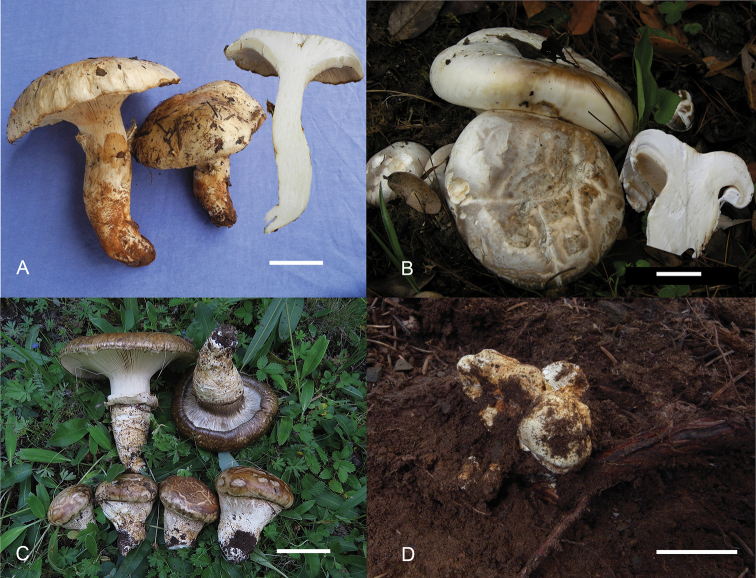
Basidiomes of *Catathelasma* species in China. **A***Catathelasmalaorentou* (HKAS 92245) **B***Catathelasmasubalpinum* (HKAS 67751) **C***Catathelasmaimperiale* (HKAS 79952) **D** Young *Catathelasmaimperiale* (HKAS 84299) in association with roots of *Piceaasperata* Mast. Scale bars: 2.5 cm.

#### Catathelasma laorentou

Taxon classificationFungiAgaricalesTricholomataceae

Z.W. Ge sp. nov.

B11BAE88-ED0B-587A-9177-2192454467DF

830871

Figs 2A , 3

##### Diagnosis.

This species is distinguished from *C.ventricosum* (Peck) Singer by having pale yellow to greyish-yellow basidiomes, longer stipes, abundant clamp connections and associations with *Pinusyunnanensis* Mast. and *Keteleeriaevelyniana* Franchet in south-western China.

##### Type.

China. Yunnan Province: Chuxiong, Zixi Mountain, alt. 1950 m, in forest dominated by *K.evelyniana* Mast. and *P.yunnanensis* Franchet, 26 August 2017, Z. W. Ge 4070 (Holotype: HKAS 105984). GenBank accession numbers: – ITS, MK909107.

##### Description.

Pileus 10–24 cm broad, hemispherical to convex at first, expanding to convex to broadly convex with age; surface initially white, then yellowish-white (1A2) to pale yellow (1A3), greyish-yellow (2B3) with age, smooth at first, irregularly depressed, margin more or less incurved, slightly viscid to viscid when wet, occasionally with whitish veil remnants. Lamellae decurrent, white to off-white when young, whitish when mature, thick, 7–15 mm in height, with 1–2 series of lamellulae, edge smooth. Stipe 6–24 × 1.5–8 cm, fusiform, attenuate downwards, straight or curved, firm, with double annulus in which the lower annulus is flimsy and the upper annulus is membranous to leathery, yellowish-white, often split into several pedals. Context white, 2.1–4.5 cm thick in pileus, white in pileus and stipe, not changing colour when cut; smell and taste farinaceous. Spore print white.

Basidiospores [70/3/3] (8) 9–12(15) × (4) 5–6.5 (7) μm (mean 9.9 ± 1.3 × 5.8 ± 0.5 μm), Q = (1.23) 1.33–2.2 (2.75), Qav = 1.72 ± 0.30, ellipsoid, oblong to subcylindrical in frontal view, subcylindrical to somewhat inequateral in side view, hyaline in KOH, amyloid, congophilous, smooth, thin walled, without germ pore. Basidia 38–50 × 8–10 μm, narrowly clavate, 4-spored, hyaline; sterigmata up to 6 μm long. Cheilocystidia basidiole-like, hyalinous. Pleurocystidia absent. Lamella trama subregular, somewhat bilateral towards lamella edge, made up of more or less parallel to interwoven hyphae. Oleiferous hyphae present in both lamella and pileus trama. Pileipellis a thick ixocutis (850–1000 μm thick) of loosely interwoven cylindrical, gelatinised hyphae 2–10 μm in width, interspersed with oleiferous hyphae. Clamp connections present, common.

##### Distribution.

Known from Sichuan and Yunnan provinces in south-western China.

##### Ecology.

Presumably ectomycorrhizal, solitary or scattered, rarely in small clusters of 2–5 basidiomes in *Pinus* or *Keteleeria* forests.

##### Etymology.

From ‘lao ren tou jun’, a transliteration of the Chinese name “老人头菌” which is a local common name used in the wild mushroom markets in Yunnan, China. The literal translation is “fungus that looks like the shiny bald pate of The God of Longevity”.

##### Additional specimens examined.

China. Yunnan Province, Chuxiong, Nanhua, wild mushroom market, 12 August 2014, Z. W. Ge 3620 (HKAS 84458); Dali, Bingchuan, Jizu Mountain, alt. 2350 m, 4 August 2013, J. Qin 728 (HKAS 81166); Kunming, Aziying, 15 August 2015, Z. W. Ge 3765 (HKAS 92245); Kunming, Yeyahu, alt. 2000 m, 22 September 2012, L. H. Han 23 (HKAS 78582); Lijiang, Ninglang, alt. 2300 m, in *Pinusyunnanensis* forest, 6 August 2011, T. Guo 368 (HKAS 71264); Sichuan Province: Muli, Liziping, alt. 2500 m, in *Pinusyunnanensis* forest, 31 July 2012, Y. J. Hao 688 (HKAS 76346).

##### Discussion.

*Catathelasmalaorentou* is morphologically similar to *C.ventricosum* (Peck) Singer, a species originally described from North America. Both species have ellipsoid basidiospores, large-sized hemispherical pilei and a pileipellis composed of an ixocutis layer. However, *C.laorentou* has abundant clamp connections, smaller basidiospores (9–11 × 5–6 μm), larger basidia (38–50 × 8–10 μm) and is found in coniferous forest dominated by *P.yunnanensis* and *K.evelyniana* from south-western China, while *C.ventricosum* is found alongside hardwood (Singer 1940).

*Catathelasmasingeri* Mitchel & A.H. Sm. from the USA is morphologically similar to *C.laorentou*, but the former differs by its dull pale ochraceous to dingy olive buff pileus which is slimy viscid and shows similarities to *Hygrophorus* Fr., smaller basidiomes (pileus around 6 cm, stipe 4 × 1.2 cm), bearing basidiole-like or narrower cheilocystidia. *Catathelasmasingeri* was collected from the aspen zone, which was dominated by *Populustremuloides* and Pinaceae species, although the specific host tree was not mentioned (Mitchel and Smith 1978).

*Catathelasmaimperiale*, originally described from Europe, is distinguished by its greyish-brown, reddish-brownish or brown basidiomes (Fig. 2C, D), cylindrical cheilocystidia with yellow contents and its association with species of *Pinus*, *Picea* and *Abies* (Læssøe and Petersen 2019; Vellinga 1999; personal observation by the first author).

**Figure 3.**
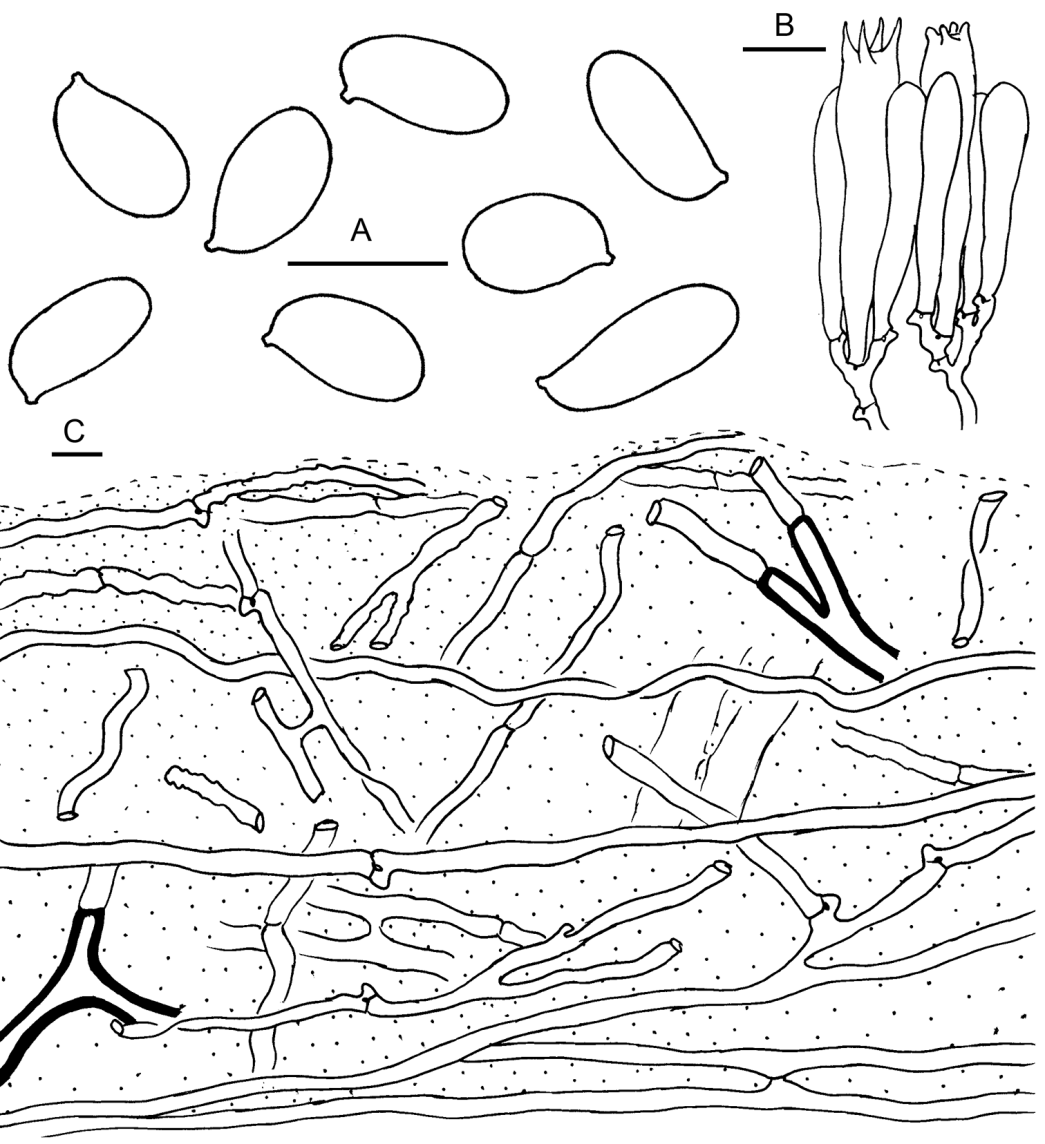
Microscopic features of *Catathelasmalaorentou* (HKAS 105984) **A** Basidiospores **B** Basidia **C** Pileipellis. Oleiferous hyphae are indicated by thick-walled hyphae. Scale bars: 10 μm.

#### Catathelasma subalpinum

Taxon classificationFungiAgaricalesTricholomataceae

Z. W. Ge sp. nov.

851BE553-C1EA-591C-B763-EECDF107494A

830872

Figs 2B , 4

##### Diagnosis.

*Catathelasmasubalpinum* is distinguished from *C.laorentou* by having greyish-yellow to grey pilei, higher elevation (alt. 2600–3500 m) occurrence and association with *Pinusdensata* Mast.

##### Type.

China. Yunnan Province: Lijiang, Ninglang, Xichuan Xiang, 14 July, 2010, J. Qin 65 (Holotype: HKAS 67751). GenBank accession numbers: – ITS, MK909099; LSU, MK909121.

##### Description.

Pileus 3.5–15 cm broad, hemispherical at early stage, expanding to broadly convex with age, shallowly depressed at centre, white to dirty white at first, then greyish-white (1B1) to greyish-yellow (4C4), grey (8B1) when mature, with incurved margin, viscid when wet, sometimes irregularly cracked. Lamellae slightly decurrent, crowded, whitish, thick, 8 mm in height, with 2–3 tiers of lamellulae, with smooth edge, covered by a white, well developed, thick membranous veil in early stage. Stipe 11–14 × 3–5.5 cm, fusiform, attenuated downwards, whitish to yellowish-white, firm, with double annulus in which the lower annulus is flimsy and the upper one is membranous, thick, around 2.5 cm away from the stipe apex; with white inner side and greyish-yellow outer side. Context white in pileus and stipe, not changing colour when cut, 3.5 cm thick in pileus; smell and taste farinaceous. Spore print white.

Basidiospores [43/2/2] (9) 10–12 × 5–6 μm (mean 10.7 ± 0.8 × 5.4 ± 0.5 μm), Q = (1.67) 1.80–2.20 (2.40), Qm = 1.99 ± 0.18, subcylindrical in frontal view, subcylindrical to somewhat inequilateral in side view, hyaline in KOH, amyloid, smooth, thin-walled. Basidia 35–45 × 8–9 μm, narrowly clavate, 4-spored; sterigmata up to 5 μm long. Pleurocystidia none. Cheilocystidia basidiole-like, hyalinous. Lamella trama subregular, somewhat bilateral towards lamella edge, made up of more or less parallel to interwoven hyphae. Oleiferous hyphae present in both lamella and pileus trama. Pileipellis a thick ixolattice (500–650 μm thick) of 1.5–10 μm wide hyphae which gelatinise and collapse, occasionally interspersed with oleiferous hyphae; the layer grading gradually into pileal trama. Clamp connections abundant in all tissues.

##### Distribution.

Known from Yunnan Province, south-western China.

##### Ecology.

Presumably ectomycorrhizal, in *Pinusdensata* forests distributed at around alt. 2600–3500 m. Solitary to scattered, terrestrial.

##### Etymology.

The epithet "*subalpinum*" refers to the distribution range of the species.

##### Additional specimens examined.

China. Yunnan Province: Lijiang, Elephant Hill, 1 August 2011, Q. Cai 495 (HKAS 70091); Ninglang, 6 August 2011, L. P. Tang 1459 (HKAS 69920).

##### Discussion.

*Catathelasmasubalpinum* is closely related to *C.laorentou*, which is also from south-western China. However, *C.subalpinum* differs by its higher elevation distribution and its association with *Pinusdensata*, while *C.laorentou* has pale yellow to greyish-yellow basidiomes, associations with *P.yunnanensis* and *Keteleeriaevelyniana* forests and is comparatively more common than *C.subalpinum*. Besides, *C.subalpinum* has much fewer oleiferous hyphae in the pileipellis. In addition, phylogenetic trees, reconstructed from ITS, 28S, TEF1 and concatenated ITS-LSU-TEF1, support the separation of *C.subalpinum* from *C.laorentou*.

*Catathelasmasubalpinum* is also morphologically similar to *C.ventricosum* Peck) Singer in general appearance. However, *C.subalpinum* is found in coniferous forest dominated by *Pinusdensata* in south-western China, while *C.ventricosum* is associated with hardwood trees in south-eastern North America (Singer 1940); *C.subalpinum* has abundant clamp connections in all tissues and longer stipes measuring 11–14 × 3–5.5 cm (compared to the 4–5 × 4 cm for *C.ventricosum*).

*Catathelasmasingeri* from USA is morphologically somewhat similar to *C.subalpinum*. However, *C.singeri* has a slimy viscid pileus that is more similar to species within the genus *Hygrophorus* Fr. (Mitchel and Smith 1978), smaller basidiomes (pileus around 6 cm, stipe 4 × 1.2 cm) compared with those of *C.subalpinum* (pileus up to15 cm, stipe 11–14 × 3–5.5 cm) and narrow, basidiole-like cheilocystidia.

*Catathelasmaevanescens*, which was described from Wyoming (USA), is similar in general appearance and also has a high elevation distribution. However, *C.evanescens* has obvious distant lamellae, a hollow stipe, a volva-like veil around the base of the stipe and longer but narrower basidiospores measuring 14–17.5 × 3–5 μm, according to Lovejoy (1910).

**Figure 4.**
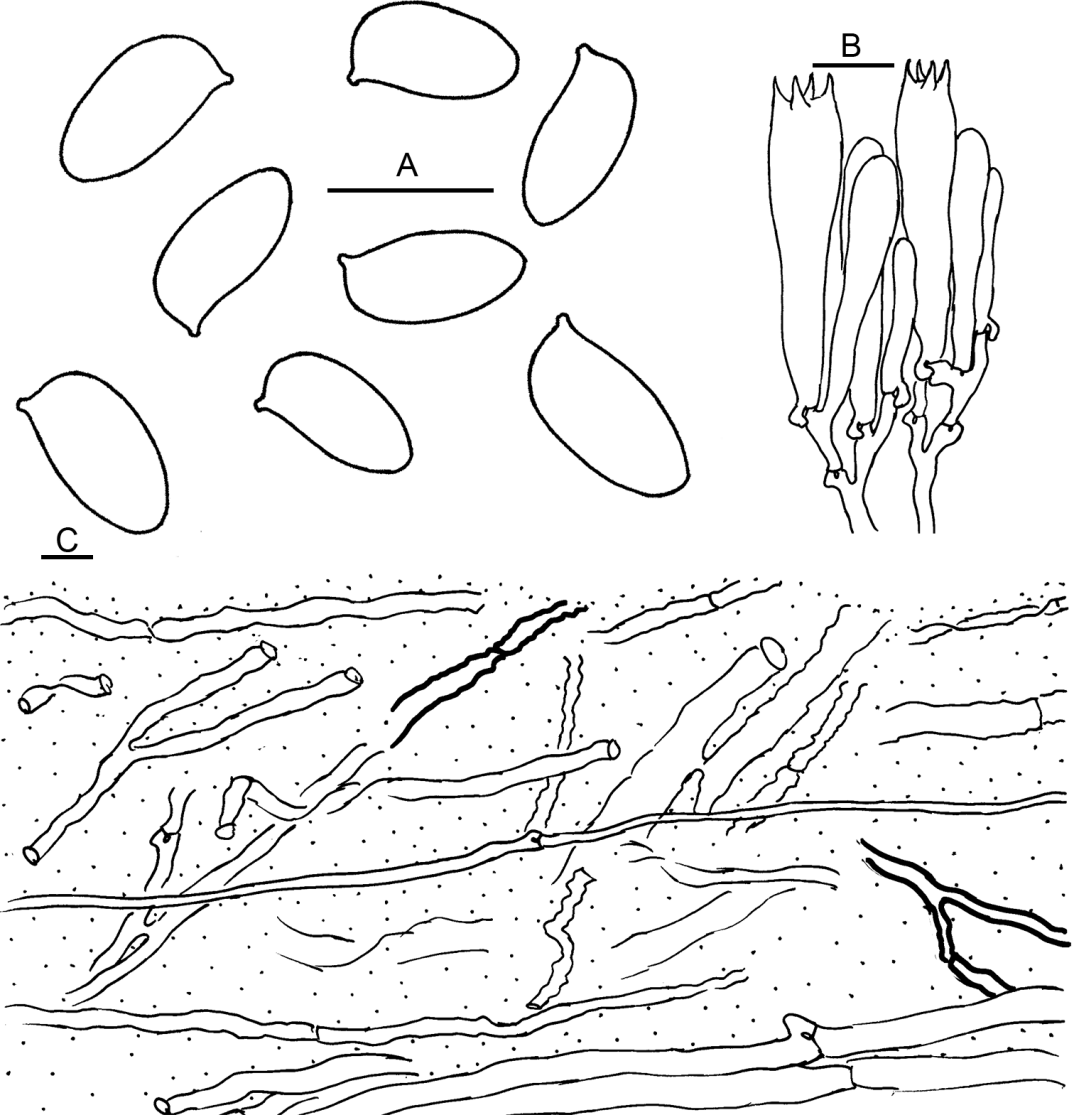
Microscopic features of *Catathelasmasubalpinum* (HKAS 67751). **A** Basidiospores **B** Basidia **C** Pileipellis. Oleiferous hyphae are indicated by thick-walled hyphae. Scale bars: 10 μm.

### Key to the known species of *Catathelasma* in China

**Table:** 

1	Pileus overall ochraceous, greyish-brown or reddish-brownish, lamellae with cylindrical or basidiole-like, yellow to brown cheilocystidia, under *Pinus*, *Piceaabies*, *Picea* spp. or *Abies* spp	***C.imperiale***
–	Pileus overall whitish, greyish-white, greyish-yellow or grey with age and sun exposure, sometimes brown in the centre, cheilocystidia basidiole-like, hyalinous, associated with *Pinus* spp. or *Keteleeria* spp	**2**
2	Pileus pale yellow to greyish-yellow, in forest dominated by *Pinusyunnanensis* or *Keteleeriaevelyniana* in lower elevation (alt. 700–2900 m)	***C.laorentou***
–	Pileus greyish-yellow to grey, in forest dominated by *Pinusdensata* Mast. in higher elevation (alt. 2600–3500 m)	***C.subalpinum***

## Discussion

### Host species and geographic distribution as important indicators in delimiting species within *Catathelasma*

Most of the characters used to identify fungal species are based on the morphology of basidiomes. However, the use of morphological characters to delimit species boundaries may be inadequate due to the paucity of measurable characters as basidiomes only represent a single and transient part of the fungal life cycle (Petersen and Hughes 1999) and this turns out to be the case in Catathelasmataceae species. In a recent study, species within *Guyanagarika* were found to be very difficult to distinguish from each other, based on morphology and recognition of species within *Guyanagarika* is only possible through molecular markers (Sánchez-García et al. 2016). Here, besides the ecological niches, the two new species that are described only differ from each other in subtle characters, such as the colours of the pileus and the density of oleiferous hyphae in the pileipellis.

Based on stable isotope evidence, *Catathelasma* is ectomycorrhizal (Kohzu et al. 1999). Indeed, habitats of known *Catathelasma* species are all in ectomycorrhizal vegetations. For example, *C.evanescens* is found in “open balsam and spruce wood” (Lovejoy 1910) and *C.imperiale* is found in forests of *Piceaabies* or other species of *Picea*, *Abies* or *Pinus* (Læssøe and Petersen 2019; Vellinga 1999). Similarly, *C.singeri* is from the aspen zone, which is dominated by *Populustremuloides* and Pinaceae species (Mitchel and Smith 1978), while *C.ventricosum* was recorded growing with hardwood (Singer 1940). *Catathelasma* sp. is reported associating with conifers such as *Piceasitchensis* (e.g. Desjardin et al. 2014, as *C.ventricosum*).

In China, *Catathelasmaimperiale* is distributed in alpine regions in western and south-western provinces, associated with *Picea* such as *Piceaasperata* or *Abies* spp. The finding of two new *Catathelasma* species in China viz., *C.subalpinum* associated with *P.densata* and *C.laorentou* associated with *P.yunnanensis* and/or *K.evelyniana*, demonstrated that species in *Catathelasma* probably possess host tree preferences, indicating a much narrower distribution than previously thought (e.g. the idea that *C.imperiale* and *C.ventricosum* are widely distributed in the Northern hemisphere). Thus, in addition to morphological characters, host tree species and geographic distribution can be of help in delimiting species within ectomycorrhizal genera such as *Catathelasma*. Indeed, mycorrhizal host association and geographic separation could contribute to fungal speciation as host-shift events can provide ecological opportunities for the diversification of ectomycorrhizal fungi (Cui et al. 2016, 2018; Han et al. 2018; Sato et al. 2017; Sánchez-García et al. 2016).

### Distribution pattern, evolutionary relationships within *Catathelasma* and future directions

Our study revealed that the geographical distribution differs amongst species of the genus: the previous records of *C.ventricosum* from China were based on incorrect identifications of *C.laorentou* or *C.subalpinum*, whereas *C.imperiale*, originally described from Europe, is indeed present in East Asia. *Catathelasmalaorentou* and *C.subalpinum* seem to be endemic to south-western China (possibly East Asia), while *C.ventricosum*, *C.evanescens*, *C.singeri* and *C.* sp. seem to be endemic to North America, but more sampling is needed to confirm these assumptions.

The phylogeny of *Catathelasma* in this study, inferred from ITS, LSU and TEF1 data, revealed that this genus contains two major clades: the /subalpinum clade and the /imperiale clade (Fig. 1). Within the /subalpinum clade, the North American species *C.* sp. (labelled as *C.ventricosum* or *C.imperiale* in GenBank) is sister to the clade jointly formed by Asian species *C.laorentou* and *C.subalpinum*.

*Catathelasmaevanescens* is considered rare and has seldom been collected since it was described. Although efforts have been made to include *C.evanescens* in the present study by sequencing the specimens identified as *C.evanescens* (DBG 6151 and DBG 21378), molecular analysis revealed that they are conspecific with *C.singeri*. To better understand the species relationships and historical biogeography of this genus, recollecting specimens from the type locality of *C.evanescens* and *C.ventricosum* is necessary. Further studies that include these two North American species and the undescribed species *C.* sp. (Fig. 1) in a multigene phylogeny are needed.

## Supplementary Material

XML Treatment for Catathelasma imperiale

XML Treatment for Catathelasma laorentou

XML Treatment for Catathelasma subalpinum
